# *In vivo* RNAi screen identifies NLK as a negative regulator of mesenchymal activity in glioblastoma

**DOI:** 10.18632/oncotarget.3980

**Published:** 2015-05-19

**Authors:** Jason K. Sa, Yeup Yoon, Misuk Kim, Yeonghwan Kim, Hee Jin Cho, Jin-Ku Lee, Gi-Soo Kim, Suji Han, Woon Jin Kim, Yong Jae Shin, Kyeung Min Joo, Patrick J. Paddison, Tohru Ishitani, Jeongwu Lee, Do-Hyun Nam

**Affiliations:** ^1^ Graduate School of Health Science & Technology, Samsung Advanced Institute for Health Science & Technology (SAIHST), Sungkyunkwan University, Seoul, Korea; ^2^ Samsung Biomedical Research Institute, Samsung Medical Center, Seoul, Korea; ^3^ Institute for Refractory Cancer Research, Samsung Medical Center, Seoul, Korea; ^4^ Department of Stem Cell Biology and Regenerative Medicine, Lerner Research Institute, Cleveland Clinic, Cleveland, OH, USA; ^5^ Human Biology Division, Fred Hutchinson Cancer Research Center, Seattle, WA, USA; ^6^ Division of Cell Regulation Systems, Medical Institute of Bioregulation, Kyushu University, Fukuoka, Japan; ^7^ Department of Neurosurgery, Samsung Medical Center, Sungkyunkwan University School of Medicine, Seoul, Korea

**Keywords:** glioblastoma, RNA interference screen, Nemo-like kinase, stemness, mesenchymal

## Abstract

Glioblastoma (GBM) is the most lethal brain cancer with profound genomic alterations. While the *bona fide* tumor suppressor genes such as *PTEN, NF1*, and *TP53* have high frequency of inactivating mutations, there may be the genes with GBM-suppressive roles for which genomic mutation is not a primary cause for inactivation. To identify such genes, we employed *in vivo* RNAi screening approach using the patient-derived GBM xenograft models. We found that Nemo-Like Kinase (NLK) negatively regulates mesenchymal activities, a characteristic of aggressive GBM, in part via inhibition of WNT/β-catenin signaling. Consistent with this, we found that NLK expression is especially low in a subset of GBMs that harbors high WNT/mesenchymal activities. Restoration of NLK inhibited WNT and mesenchymal activities, decreased clonogenic growth and survival, and impeded tumor growth *in vivo*. These data unravel a tumor suppressive role of NLK and support the feasibility of combining oncogenomics with *in vivo* RNAi screen.

## INTRODUCTION

Glioblastoma (GBM) is the most common and lethal primary brain tumor [1]. Unfortunately, the current standard-of-care for GBM patients provides only palliation with a median survival of 14.6 months in spite of surgery, chemotherapy, and radiation [2, 3]. Molecular circuits and factors behind its aggressive behaviors and malignant characteristics remain incompletely understood. One of the key components for GBM malignancy is the loss of functional tumor suppressor genes. Tumor suppressors act in signaling networks that restrict cellular proliferation and present barriers to malignant transformation. Even though tumor suppressor genes *per se* may not be considered optimal as drug targets, their loss of function could create cellular dependency which could be exploited therapeutically [4].

Tumor suppressors are disabled by combinations of point mutations, genomic deletions and promoter methylation. Indeed, recent large-scale genomic studies on patient-derived GBM specimens conclusively showed various genomic copy number alterations and mutations [5, 6]. For example, genomic deletions and mutations of *TP53, NF1*, and *PTEN* are frequently found in various cancers including GBMs. Because mutations in one allele are often followed by deletion of the other, somatic deletions in human cancers often pinpoint tumor suppressor genes that function as ‘drivers’ of tumor evolution. However, the large-scale genomic analyses also revealed the large list of genes that may have “tumor-suppressive” roles but the frequencies of inactivating mutations are relatively uncommon [6]. We set out to determine the functional roles of these candidate genes in gliomagenesis.

By combined *in silico* analyses of genomic copy variation (CNV) and transcriptome profiling of human GBM specimens, we have derived the gene sets whose genome copy numbers and expression levels are significantly low in GBM specimens. To interrogate the functional roles of the candidate genes in a relevant and systemic manner, we have adapted stable RNA interference (RNAi) screening technology *in vivo*. By implementing loss-of-function genetics in the setting of *in vivo* GBM models that mimic the biology of human GBM, we validated the effects of our candidate genes on tumor growth. Here, we have identified NLK as a putative tumor suppressor gene and demonstrated that NLK plays a critical role in tumor restriction through regulation of Wnt/beta-catenin pathway and mesenchymal activity in GBM.

## RESULTS

### *In vivo* RNAi screen utilizing human GBM-derived xenograft models

To identify putative tumor suppressor genes in GBM, we first generated the candidate gene sets by utilizing genomic and transcriptome data of patient-derived GBM specimens (*n* = 228) publicly available from Rembrandt. We selected candidate genes by the levels of genomic deletions and low mRNA expressions in tumors compared to non-tumor brains (*n* = 28). The cut-off for genomic deletion was less than 1.6 of genomic copy number (compared to 2.0 in normal cells) in more than 15% of the GBM specimens. Differential expression of a given gene in GBM and non-tumor brains was determined by Affymetrix array data and statistically validated. Through TCGA and cBioPortal database, we determined the reported frequency of somatic mutations (Figure S1). As expected, these candidate gene sets include well-known tumor suppressor genes, *PTEN* and *CDKN2A/B* (Table S1). Loss of heterozygosity (LOH) on chromosome 10 is known to be the most frequent genetic alteration in GBMs and it has been suggested that multiple tumor suppressor genes may exist on chromosome 10 [7]. Consistent with this, majority of the candidate gene sets were located on chromosome 10. We have generated a shRNA pool directed against these gene sets by selecting individual shRNA lentiviral clones from Cold Spring Harbor shRNA libraries. On average, there were 5 to 7 shRNA clones for each targeted genes.

**Figure 1:**
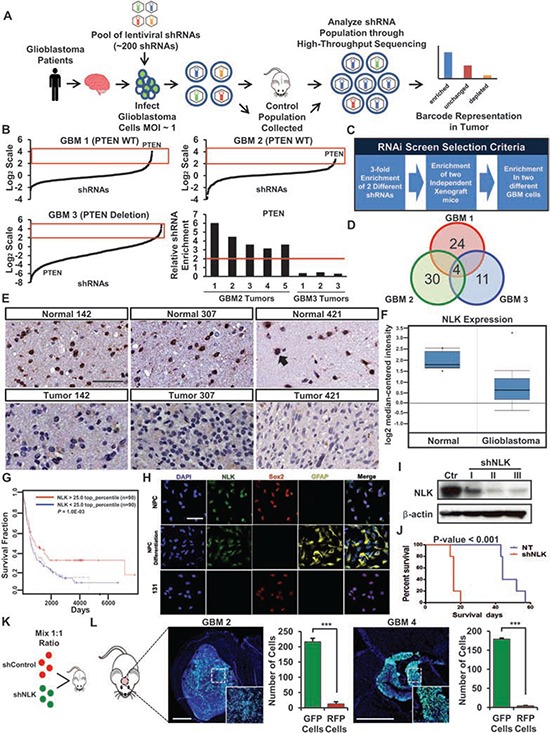
*In vivo* RNA interference screening identifies putative tumor suppressors in GBM **A.** Schematic representation of *in vivo* RNAi screening. Patient-derived GBM cells are transduced with the shRNA library pool and injected into the mice brains. Tumors were harvested and shRNAs were PCR amplified and deep sequenced to identify candidate “hits”. **B.** Analysis of the shRNAs recovered from our RNAi screening. Data are normalized to the Control population and plotted in Log2 scale. Red box indicates enriched shRNAs. Enrichment of PTEN shRNAs in GBM 2 tumors (PTEN WT) compared to the GBM 3 tumors (PTEN deletion). **C.** A selection criteria for candidate hits from the RNA interference screen. **D.** Candidate “hits” from the screen. **E.** Immunohistochemistry of NLK on TissueMicroArray (TMA) containing 88 GBM samples and 32 normal brain samples. Scale bar, 100 μm. **F.** Oncomine microarray data analysis for NLK expression in glioblastoma versus normal brain tissues. (*p* < 0.001, *n* = 525) **G.** Analysis of REMBRANDT public dataset on glioma patient survival in accordance with NLK^high^ and NLK^low^ expressions (*n* = 90 each). **H.** Representative confocal microscopy images of immunofluorescence (IF) staining of NLK, Sox2, and GFAP in normal neural progenitor cells (NPC), differentiated NPC, and GBM cells. Scale bar, 100 μm. **I.** Immunoblots of NLK in patient-derived GBM cells transduced with Control or shRNA NLK. **J.** Kaplan-Meier survival curves of Control vs shRNA NLK. **K–L**, Schematic representation of dual-color competition assay *in vivo*. K A total of 50,000 cells from a 1:1 mixture of RFP-labeled shControl cells (red) and GFP-labeled shNLK cells (green) were implanted into mouse brains. L Immunofluorescence images of cryo-sectioned mouse brains. Scale bar, 500 μm. Bar graph represents the number of GFP and RFP positive cells that were counted in different spots of the tumor that were selected randomly. (+ SD, *n* = 4).

Our experimental scheme for *in vivo* RNAi screen was summarized in Figure 1A. We aimed to achieve that each tumor cell would be integrated with a single unique shRNA clone. Once these cells were injected for orthotopic tumor generation in mouse brains, a subset of tumor cells would outgrow presumably due to the selective growth advantages conferred by a specific shRNA (Figure 1A). As each shRNA vector was uniquely labeled with a DNA barcode, sequencing analysis of the resultant tumors will inform the relative contribution of each clone in tumor. By using GFP expressing shRNA vectors, we determined the optimal multiplicity of infection (MOI) to ensure that most cells would intake a single copy of the lentiviral shRNA (data not shown).

We have previously established a series of patient-derived GBM xenografts and demonstrated that these tumors maintain the genomic and biological characteristics of the parental GBM tumors [8]. In these GBM models, intracranial injection of 100, 000 tumor cells was sufficient to generate tumors with near 100% tumor take efficacy. Complexity of shRNA pools and the number of different shRNAs in a given population are crucial factors of *in vivo* RNAi screen [9]. Through titration and cell tracing analyses, we determined that an average representation of approximately 500 cells per shRNA construct could provide a reliable index to ensure the detection of each shRNA clone and prevent any initial depletion of certain shRNAs prior to intracranial injection procedure and the overall screening process (data not shown). With these optimization processes, we have constructed the lentiviral shRNA pool containing approximately 200 individual shRNAs, transduced into patient-derived GBM cells, and injected the resulting cells into recipient mice or collected immediately as the control population. Three different patient-derived GBM cells (GBM 1 to 3, 5 mice for each GBM) were used in this screen to cover the diverse GBM phenotypes. By using ArrayCGH and Whole Exome Sequencing, we characterized genomic profiles of these tumors. All three GBMs contain homozygous genomic deletion of *CDKN2A*. GBM 1 and 2 harbor wild-type *PTEN* gene, while GBM 3 does not express any PTEN proteins. Once body weight loss and changes in health status including neurological signs have been observed, xenograft tumors were harvested for the extraction of tumor DNA. After PCR amplification of shRNA hairpins from tumor-derived genomic DNA, hairpin representations were analyzed using high-throughput sequencing technology.

We have acquired a list of shRNA hairpins that are enriched for more than 3-fold in xenograft tumor cells compared to the initial control populations (Figure 1B). To avoid false positive results derived by random accumulation of specific shRNAs and/or potential off-target effects, we set additional criteria for the enriched candidate genes; 1) at least 2 different shRNAs should be detected for a single gene, 2) the enriched shRNAs should be observed in at least two independent xenograft tumors, and 3) the enriched shRNAs should be observed in at least two different GBM cells (Figure 1C). PTEN-shRNA harboring cells were enriched for more than 5 fold in GBM1 and GBM2 but not in GBM3 (Figure 1B). As predicted considering homozygous genomic deletion of *CDKN2A* in these tumors, *CDKN2A* shRNAs were not enriched in any screens. Collectively, these results support the fidelity of our *in vivo* RNAi screen and validation procedures.

In addition to *PTEN, in vivo* RNAi screens further identified 4 candidate genes, including MXI1, NLK, and MAPK8 (Figure 1D). As previous studies have shown that MXI1 and PTEN were both involved in GBM as tumor suppressor genes for their abilities to regulate tumor cell growth, migration, and apoptosis [10–13], we decided to focus on NLK. Anti-tumorigenic effects of NLK have been characterized in different cancer types [14–16], but relatively little is understood about the role of NLK in GBM. While NLK has been implicated in Wnt signaling pathway, a critical regulator in GBM [17], it remains unclear how NLK-WNT signaling axis affects stem cell associated phenotypes or whether NLK loss is specifically linked to GBM subtypes.

First, we determined the expression levels of NLK protein in matched GBM and adjacent non-tumor brain specimens by tissue microarray (TMA). In contrast to high NLK expression in non-tumor tissues, GBM tissues have little or no expression (Figure 1E). Quantitation of immunohistochemical analyses using the sections derived from 88 GBM specimens and non-tumor brain tissues revealed significant difference in NLK expression. To further validate these findings, we surveyed the expression levels of NLK mRNA in glioma specimens utilizing Oncomine and Rembrandt databases [18, 19] and found that NLK mRNA levels are significantly lower in GBMs compared to non-tumor brain tissues (Figure 1F). When we stratified glioma patients by NLK mRNA levels, a subset of glioma patients with top 25% of NLK mRNA survived significantly longer than the remaining patients, suggesting a potential association between NLK levels and patient survival (Figure 1G).

As NLK is expressed in normal brain tissues, we further determined expression of NLK in normal neural progenitor cells (NPCs) and their differentiated progenies. Regardless of their differentiated status, SOX2-positive NPCs and astrocyte marker GFAP-positive cells both expressed NLK. In contrast, the patient-derived GBM cells (131) displayed little or no NLK expression (Figure 1H).

Some GBM specimens including the samples that were used for *in vivo* RNAi screen express NLK, albeit much lower compared to normal brain tissues, as shown in Figure 1F. To validate RNAi screening results, we further inhibited NLK by shRNA-mediated knockdown and evaluated its effects on tumor growth (Figure 1I–1L). Control or NLK shRNA expressing tumor cells were implanted orthotopically and animal survival was determined. Mice injected with NLK shRNA expressing cells died much faster than the mice with control cells (Figure 1J). Additionally, we performed *in vivo* growth competition assays in which control (RFP labeled) or NLK shRNA tumor cells (GFP labeled) were mixed and co-injected into mice brains. We harvested the resulting tumors and performed FACs analysis and histological analysis. Although the same number of tumor cells was injected, more than 95% of the resulting tumor cells were derived from GFP-positive, NLK shRNA expressing cells (Figure 1L). Together, these results support that NLK inhibition facilitates *in vivo* tumor growth.

### NLK impedes clonogenic growth and stem-like properties of GBM

**Figure 2:**
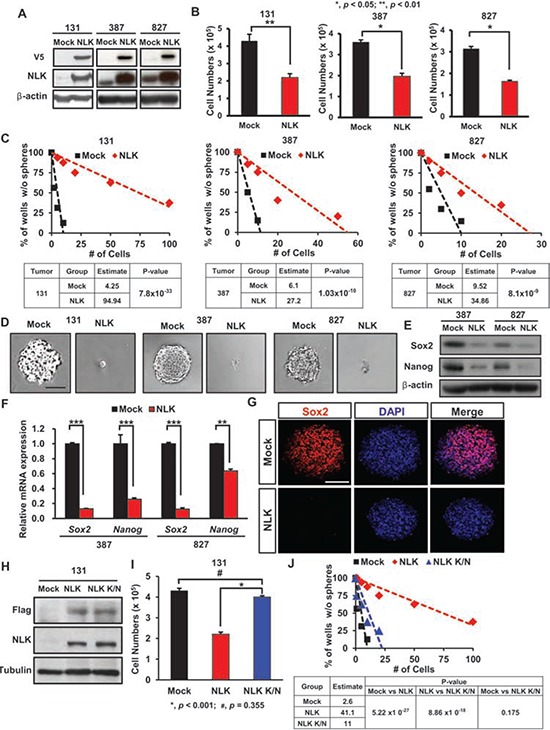
NLK regulates proliferation and stem cell-like properties in patient-derived primary GBMs **A.** Immunoblots of V5-NLK and NLK in patient-derived GBM cells transduced with Mock control or NLK-WT vector. **B.** Comparison on the effects of NLK on *in vitro* proliferation. (+SD, *n* = 5) **C.** Limiting dilution assays (LDA) for *in vitro* tumor sphere formation. LDA clonogenic significance is measured by the linear regression analysis. **D.** Overexpression of NLK suppresses tumor sphere formation. Scale bar, 100 μm. **E.** Immunoblots of Sox2 and Nanog in NLK-WT or mock transduced GBM cells. **F.** Real-time RT-PCR analysis to determine the effects of NLK on mRNA expression levels of *Sox2* and *Nanog*. **G.** Representative confocal images of immunofluorescence (IF) staining of Sox2 in spheroids. Scale bar, 100 μm. **H.** Immunoblots of anti-Flag and NLK in patient-derived GBM cells transduced with Mock control, NLK-WT or NLK Kinase-Negative (K/N) mutant vector. **I.** Comparison on the effects of NLK-WT and NLK K/N on *in vitro* proliferation. **J.** Limiting dilution assays (LDA) for *in vitro* tumor sphere formation.

To elucidate the function of NLK in GBM, we first investigated its ability to regulate cell proliferation in GBM by ectopic expression of NLK. Three patient-derived GBM cells 387, 827, and 131 express low levels of NLK proteins. Transduction with lentivirus expressing NLK-wild type (WT) significantly increased the expression of NLK in these cells (Figure 2A). Short-term proliferation kinetics of tumor cells, determined by MTT assays, demonstrated a significant decrease of cell proliferation in NLK-transduced GBM cells compared to the control groups (Figure 2B).

Cancer stem/initiating cells (CSCs) are functionally defined by their enhanced ability to initiate tumors *in vivo*. Although some cancers may not follow the CSC model, numerous studies support that GBMs harbor a subpopulation of highly tumorigenic, stem-like cells (GSCs) [20–23]. GSC population is enriched with self-renewal capacity, which may contribute to the aggressive behavior of GBM [24–26]. Given an anti-proliferative effect of NLK, we reasoned that NLK could influence stem-like properties of GBM. Clonogenic growth of GBM cells as spheroids is an indicator of stem-like GBM cells *in vitro*. We plated tumor cells at the clonal density ranging from 1 to 50 cells per well and monitored clonogenic growth of tumor cells with or without overexpression of NLK. The estimated frequencies of clonogenic cells were significantly high in the control tumor cells (1/4, 1/6, and 1/9 for 131, 387, and 827 cells, respectively) than NLK-overexpressing cells (1/95, 1/27, and 1/35 for 131, 387, and 827 cells, respectively), implicating the role of NLK in clonogenic growth of GBM (Figure 2C and 2D). To further corroborate the association between NLK and stemness, we determined the expression levels of well-known GSC-associated factors such as Nanog and Sox2. Results from real-time RT-PCR, immunoblot, and immunofluorescence analysis revealed significantly low levels of Nanog and Sox2 in NLK-overexpressing cells compared to the control cells (Figure 2E–2G). Together, these results indicate a negative role of NLK in GBM clonogenic growth and stem cell-like properties.

Previous studies showed that NLK-mediated phosphorylation is critical for its downstream effectors [17, 27, 28]. To examine whether tumor-suppressive role of NLK is dependent on kinase activity, we overexpressed NLK kinase-inactive mutant (NLK K/N) in GBM cells and determined its effects on short-term proliferation and clonogenic growth of GBM cells. Results from the MTT assays and limiting dilution sphere forming assays indicated that NLK K/N is ineffective to elicit NLK-mediated responses (Figure 2H–2J), indicating that the tumor-suppressive role of NLK in GBM is through its kinase activity-dependent manner.

Although 387, 827, and 131 GBM cells have low levels of endogenous NLK, a subset of GBM cells have relatively high levels of NLK, reflecting GBM heterogeneity (Figure S2A). To determine the role of NLK in this subset of GBM, we performed *NLK* shRNA-mediated knockdown experiments (Figure S2B and S2C). Knockdown of NLK significantly increased clonogenic growth of 047T GBM cells. Expression levels of Nanog and Sox2 were higher in NLK knockdown cells compared to the control cells. Taken together, these data suggest that NLK impedes the clonogenic growth of GBM cells and stem cell factor expressions in a gene dosage-dependent manner.

### NLK downregulates Wnt/beta-catenin signaling pathway activation

**Figure 3:**
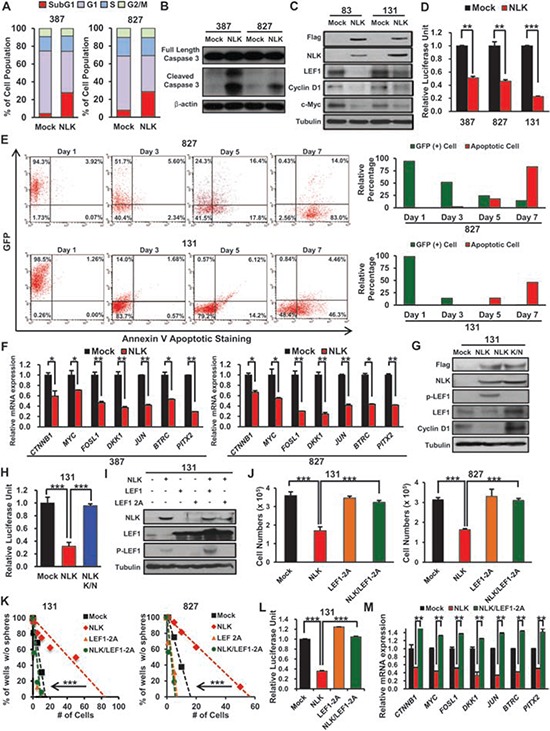
NLK negatively regulates Wnt signaling pathway and its downstream targets **A.** Mock and NLK-WT cells have been stained with PI and cell cycle was examined by flow cytometry. *P*-values were calculated using the Fisher's exact test (*p* < 0.01). **B.** Immunoblots of Caspase 3 in GBM cells transduced with Mock or NLK-WT. **C.** Immunoblots of Wnt related proteins; anti-Flag, NLK, LEF1, Cyclin D1, and c-Myc. **D.** The luciferase reporter assay was used to study TCF/LEF promoter activity in Mock or NLK-WT GBM cells. (+ SD, *n* = 3). **E.** Flow cytometric analysis of GFP and apoptosis in NLK-WT GBM Cells. GBM cells were cultured and analyzed after 1, 3, 5, or 7 days post NLK-WT transduction. Bar graph represents relative GFP positive and apoptotic cell percentage in the given days. **F.** Real-time RT-PCR of NLK effects on mRNA expression levels of beta-catenin and its downstream target genes (*MYC, FOSl1, DKK1, JUN, BTRC*, and *PITX2*). **G.** Immunoblots of Wnt related proteins in mock, NLK-WT, and NLK K/N GBM Cells; anti-Flag, NLK, phosphor-LEF1, LEF1 and Cyclin D1. **H.** Relative luciferase activities of mock, NLK-WT, and NLK K/N GBM cells **I.** Immunoblots of NLK, LEF1 and phosphor-LEF1 in GBM cells transduced with mock, NLK-WT, LEF1, LEF1-2A mutant, NLK-WT/LEF1, or NLK-WT/LEF1-2A mutant. **J.** Comparison on the effects of mock, NLK, LEF1-2A mutant and NLK/LEF1-2A mutant on *in vitro* proliferation. (+SD, *n* = 5) **K.** Limiting dilution assays (LDA) for *in vitro* tumor sphere formation. **L.** Relative Luciferase activities of Mock, NLK-WT, LEF1–2A, and NLK/LEF1–2A GBM cells. **M.** Real-time PCR analysis of beta-catenin and its downstream target genes.

As we found that NLK overexpression impeded GBM proliferation and clonogenic growth, we further evaluated the effects of NLK on cell cycle kinetics and apoptosis. NLK overexpressing GBM cells also induced significant increase in sub G0/G1 populations and levels of activated caspase 3, suggesting that NLK-overexpressing cells undergo apoptosis (Figure 3A and 3B). To investigate the downstream molecular events of NLK signaling in GBM, we surveyed a few molecular effectors including WNT signaling pathway. Previous studies have shown that WNT pathway is activated in GBM and that Wnt/beta-catenin signaling in GBM contributes to the maintenance of stem-like properties including inhibition of differentiation and invasive growth pattern [29–33]. As NLK is implicated as a negative regulator of Wnt signaling by interacting with and suppressing transcriptional activity of TCF/LEF family proteins [34], we suspected that NLK negatively regulates Wnt signaling pathway activity in GBM as well. To test this hypothesis, we overexpressed NLK in GBM cells and examined the expression levels of WNT pathway associated genes. Immunoblot analyses showed that expression levels of Wnt pathway associated proteins including LEF-1, CyclinD1, and c-Myc were significantly lower in NLK overexpressing cells compared to the control (Figure 3C). Consistent with these results, WNT responsive promoter luciferase assays showed that NLK overexpression attenuated TCF/LEF-mediated transcriptional activity of GBM cells (Figure 3D). As we observed significant apoptosis in NLK-overexpressing GBM cells, we performed time-course experiments to determine kinetics of apoptosis and WNT inhibition upon NLK overexpression. As TCF/LEF reporter expresses GFP driven by TCF/LEF binding sites, we examined the presence of GFP positive cells with Annexin V-positive apoptotic cells through Fluorescence-activated cell sorting (FACS). Control cells have high WNT activity, as most of the cells were GFP positive. Our results from two different GBM cells showed that NLK overexpression significantly decreased GFP positive cells within a day or two, while proportion of apoptotic cells significantly increased at day 5 of NLK overexpression (Figure 3E). These results suggest that inactivation of WNT pathway is a key molecular event in NLK overexpression. To further characterize the effects of NLK on Wnt pathway activation, we determined mRNA expression levels of known WNT target genes by real time RT-PCR analysis (Figure 3F). Two different GBM cells that overexpress NLK have significantly low levels of the representative WNT associated and target genes including *DKK1* and *Myc* compared to the control cells. Conversely, NLK knockdown significantly upregulated the expression of WNT pathway genes in 047T GBM cells which have a high level of endogenous NLK (Figure S3A).

As NLK suppresses growth properties of GBM through a kinase activity-dependent manner (Figure 2H–2J), we examined the effect of kinase-inactive NLK mutant on Wnt downstream pathway in 131 GBM cells. 131 cells were transduced with the empty vector control, wild type NLK, or NLK mutant-expressing lentivirus, cultured for 2 days, and harvested for immunoblot analysis. Kinase-inactive NLK mutant, in contrast to the wild type NLK, was unable to suppress Wnt pathway associated factors (Figure 3G). In addition, TCF/LEF-mediated transcriptional activity and WNT target gene expression levels were unaffected by NLK K/N (Figure 3G and 3H; Figure S3B). As LEF-1 phosphorylation by NLK is a mechanism to downregulate WNT pathway activation, we determined phosphorylation status of LEF-1 upon ectopic expression of NLK. Elevated level of phosphorylated LEF-1 was found in GBM cells with NLK but not with NLK K/N mutant (Figure 3G). To evaluate the effect of LEF-1 in NLK signaling, we performed functional rescue experiments. As shown above, NLK overexpression significantly decreased proliferation, clonogenic growth and Wnt target gene activities. However, co-expression of NLK and the LEF1-2A mutant, a constitutively active mutant that cannot be phosphorylated by NLK, completely blocked NLK-mediated changes, suggesting that LEF-1 is a key mediator for NLK signaling in GBM (Figure 3I–3M). Collectively, these data implicate that NLK negatively regulates canonical Wnt signaling pathway in primary GBM.

### NLK negatively regulates mesenchymal activity

**Figure 4:**
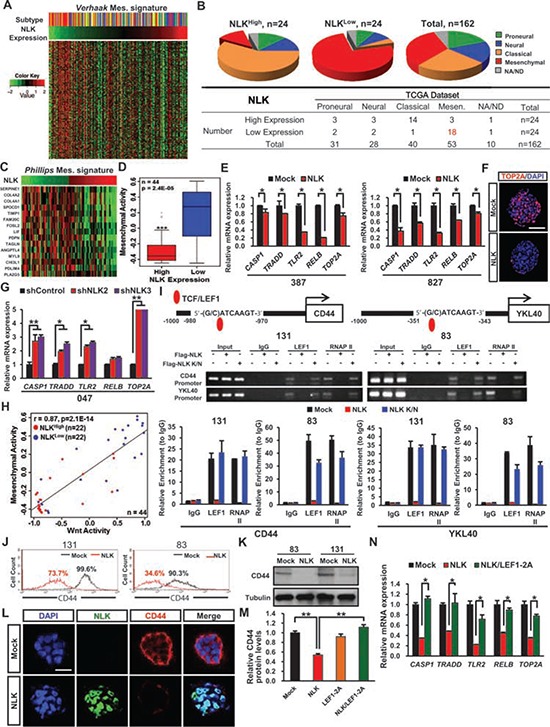
NLK influences on mesenchymal and Wnt activities **A.** Heatmap representation of *Verhaak* mesenchymal-associated gene signature profiles from the TCGA GBM RNA-seq samples according to NLK expression level. (*n* = 162). **B.** Pie-chart representation of Proneural, Neural, Classical, and Mesenchymal subtypes in TCGA GBM samples in correspondence with NLK^high^ and NLK^low^ groups. **C.** Heatmap representation of *Phillips* Mesenchyaml-associated gene signature profiles. **D.** Rembrandt microarray data analysis for Mesenchymal activity in high NLK expressing GBM patients vs. low NLK expressing GBM patients. **E.** Real-time RT-PCR analysis to determine the mRNA expression levels of Mesenchymal associated genes (*CASP1, TRADD, TLR2, RELB*, and *TOP2A*). **F.** Representative confocal microcopy images of immunofluorescence (IF) staining of TOP2A in spheroids. Scale bar, 100 μm **G.** Real-time RT-PCR analysis of Mesenchymal associated genes. **H.** Co-expression of Mesenchymal and Wnt activities in high and low NLK expressing groups. (*r* = 0.87, *p* = 2.1 × 10^−14^, Pearson correlation test). **I.** Chromatin immunoprecipitation (ChIP) analysis of LEF1 on CD44 and YKL40 promoters. **J.** Flow cytometric analysis of CD44. Gates were drawn based on isotype negative-control staining (not shown). **K.** Immunoblots of CD44 in mock and NLK-WT GBM Cells. **L.** Representative confocal images of immunofluorescence (IF) staining of NLK and CD44 in spheroids. Scale bar, 50 μm. **M.** Relative CD44 protein levels derived from quantification of immunoblot analysis using ImageJ. **N.** Real-time RT-PCR analysis of Mesenchymal associated genes.

As the recent genomic studies on large sets of GBM indicated distinct GBM subtypes based on their unique genomic alteration and gene expression patterns [35, 36], we determined the potential correlations between expression levels of *NLK* and GBM subtypes. When we stratified GBMs into four or five subtypes and determined the average NLK mRNA expression levels in each subtype, NLK expression appeared to be the lowest in mesenchymal subgroup, although proneural subtype also showed low expression levels for NLK mRNA (Figure S4A). On the other hand, more detailed analysis revealed that there are distinct subsets of GBMs that have especially low NLK mRNA expression levels. When we determined the subtype of these GBMs (*n* = 24 out of 165 GBMs), 75% of them belonged to mesenchymal subtype. In contrast, GBM subsets (*n* = 24) with the highest NLK mRNA expression among 165 GBMs, about 50% of them were identified as classical GBM subtype (Figure 4A and 4B). To further analyze the relationship between NLK expression level and mesenchymal activity, we selected NLK^high^ and NLK^low^ GBM specimens based on NLK mRNA expression levels and examined their associations with the gene sets that were used to define the mesenchymal GBM subtypes [37] as an indicator for mesenchymal activity. NLK^low^ GBMs have high mesenchymal activity whereas NLK^high^ GBMs have very low mesenchymal activity, indicating a striking inverse correlation (Figure 4C and 4D). These findings are intriguing because mesenchymal activities in GBMs are often associated with worse prognosis and more aggressive behaviors [36, 38]. To investigate whether loss of NLK may contribute to its pathology, we determined the expression levels of mesenchymal marker genes such as CASP1, TLR2, and RELB [8, 37] in GBMs with or without NLK overexpression. Ectopic expression of NLK significantly decreased expression of mesenchymal marker genes, determined by real-time PCR analysis and immunohistochemical analysis (Figure 4E and 4F). Conversely, NLK knockdown in 047T GBM cells that have high NLK expression induced expression of mesenchymal markers (Figure 4G).

In addition, the mesenchymal marker expression levels were also unaffected or elevated by NLK K/N indicating that the mesenchymal activity may be regulated through kinase-dependent manner (Figure S4B). As our data indicate WNT activation as a consequence of NLK loss, we determined a relationship between NLK and Wnt activities in these GBM specimens. As a surrogate indicator of Wnt activity activation, we used the metagene sets that were previously reported [39]. Using the gene lists used for mesenchymal subtypes, we similarly assigned the level of mesenchymal activity for each GBM as well. Strikingly, NLK^low^ GBMs have very high levels of both WNT and mesenchymal activities, while NLK^high^ GBMs have low activities (Figure 4H). Intriguingly, this inverse correlation was especially prominent in a subset of GBMs that possess high WNT activity.

To further investigate molecular association between NLK loss and mesenchymal activity, we examined transcriptional regulation of CD44 and YKL40, widely known markers of mesenchymal GBM subtype [36]. Promoter analysis of both *CD44* and *YKL40* gene revealed putative TCF/LEF binding sites. We overexpressed NLK or NLK K/N mutants in NLK low GBM cells (131 and 83) and performed chromatin immunoprecipitation-PCR (CHIP-PCR) (Figure 4I). Chromatin immunoprecipitation using anti-LEF-1 antibody using the lysates from NLK-low GBM cells pulled down the promoter DNAs of both *CD44* and *YKL40*. In contrast, CHIP-PCR results using the lysates from NLK overexpressing cells were negative, suggesting that transcription of CD44 and YKL40 in these cells at least is regulated by NLK. To validate these results, we performed FACS analysis on GBM cells with or without NLK overexpression using anti-CD44 antibody. Both 131 and 83 GBM cells have more than 90% of CD44 positive cells. Upon NLK overexpression, the number of CD44 positive cells and intensity of CD44 staining were significantly decreased (Figure 4J). Consistent with this, immunoblot analysis and immunofluorescence revealed significantly lower expressions of CD44 in NLK-overexpressing cells compared to the control cells (Figure 4K and 4L). Finally, to determine whether NLK loss regulates mesenchymal activity through LEF-1, we determined the expression levels of CD44 and other mesenchymal markers in GBM cells expressing NLK alone, LEF-1 2A mutant, or both NLK and LEF-1 2A mutant (Figure 4M and 4N). NLK overexpression inhibited the expressions of mesenchymal markers, and this inhibition was completely blocked by co-expression of the constitutively active LEF-1 2A mutant, suggesting NLK negatively regulates mesenchymal activity through LEF1. Together, these results support the notion that NLK is a critical regulator for Wnt and mesenchymal activities in GBM.

### NLK suppresses GBM tumor growth *in vivo*

**Figure 5:**
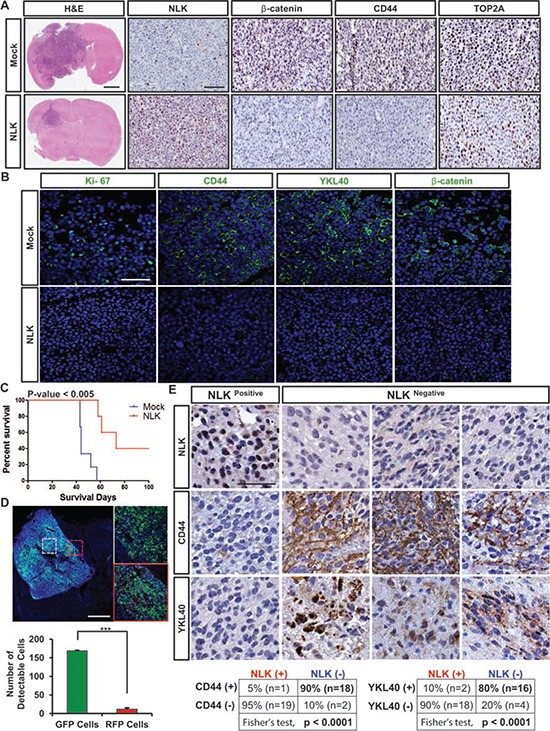
NLK overexpression down-regulates mesenchymal signature activity and impedes tumor growth *in vivo* **A.** Representative Immunohistochemical (IHC) images of hematoxylin and eosin (H&E), NLK, beta-catenin, CD44, and Top2A staining. Scale bars: H&E, 1mm;rest of the images, 300 μm. **B.** Representative Immunofluorescence images of Ki-67, CD44, YKL40, and beta-catenin staining using the frozen sections of tumor bearing brains. Scale bar, 75 μm. **C.** Kaplan-Meier survival curves of Mock vs. NLK groups. **D.** Dual-color competition assay *in vivo*. A total of 10, 000 cells from a 1:1 mixture of RFP-labeled NLK-WT cells (red) and GFP-labeled Mock cells (green) were implanted into mouse brains. Scale bar, 500 μm. Bar graph represents the number of GFP and RFP positive cells that were counted in different spots of the tumor that selected randomly. (+ SD, *n* = 4) **E.** Immunohistochemistry of NLK, CD44 and YKL40 on TMA containing 88 GBM samples and 32 normal brain samples. Scale bar, 100 μm.

The above data suggest that NLK negatively affects the proliferation and clonogenic growth of GBM cells in part via down-regulation of WNT activation and mesenchymal activity. To determine the role of NLK in tumor propagation *in vivo*, we generated intracranial xenograft tumor models derived from the control and NLK-overexpressing GBM cells. Hematoxylin and eosin (H&E) staining of mouse brain sections showed a significant difference in tumor volumes between the control and the NLK overexpression group (Figure 5A). Immunohistochemcial analysis using representative mesenchymal markers CD44 and Top2A revealed significantly low levels of these proteins in NLK-overexpressing cells (Figure 5A). Immunofluorescence staining of different GBM sections also showed the same trend (Figure 5B). These results are highly consistent with our *in vitro* data shown above and suggest that NLK suppresses *in vivo* tumor growth through negatively regulating Wnt and mesenchymal activities. Consistent with decreased proliferation index in NLK-overexpressing tumors (Figure 5B), the mice injected with NLK-overexpressing cells survived much longer (median survival 73 days) than those with the control cells (median survival 44 days, *p* < 0.005) (Figure 5C). In order to track NLK-overexpressing cells more closely *in vivo*, we used dual-color competition assays to evaluate the effect of NLK on GBM tumor propagation. Control and NLK-overexpressing GBM cells were differentially labeled with GFP- and RFP-expressing lentiviruses, respectively. These tumor cells were mixed to 1:1 mixture and co-injected into the brains of mice. More than 95% of the resultant tumor cells were GFP-positive, indicating that NLK impedes tumor propagation *in vivo* (Figure 5D).

Finally, to determine the clinical relevance of the NLK loss and mesenchymal activities, we performed TMA IHC analysis using antibodies against mesenchymal markers CD44 and YKL40 (Figure 5E). NLK^high^ and NLK^low^ GBM specimens were confirmed by NLK immunohistochemical analysis. Strong inverse correlation between NLK levels and CD44 and/or YKL40 was observed, further supporting that NLK loss is a critical regulator for high mesenchymal activities.

## DISCUSSION

Here we presented a systemic approach for identifying and validating a candidate putative tumor suppressor in GBM, which consists of the candidate gene selection based on genomic copy number alterations and mRNA expression levels of GBM specimen, *in vivo* loss-of-function screening, and subsequent validation in patient-derived primary GBM models. This approach allowed us to identify NLK as a candidate tumor suppressor and a key regulator of GBM pathogenesis. We showed that restoration of NLK impeded GBM growth *in vitro* and *in vivo*, and suppressed WNT signaling, mesenchymal activity, and stem-like features of GBM.

Recent large-scale genomic analyses, especially with high-resolution genomic sequencing and exome sequencing, have identified a large number of novel oncogenes and oncogene-associated mutations, such as IDH1 and histone 3.3 [40, 41]. In the case for genes with putative tumor suppressive role, frequencies of inactivating mutations in GBM are generally low except a few well-known bona fide tumor suppressor genes such as *p53, NF1, RB*, and *PTEN*. For example, *CDKN2A* is the well-known tumor suppressor and homozygous deletion of this gene is frequently detected in GBM, but there is little report for inactivating mutation. While promoter methylation and chromatin modification are likely associated with inactivation of putative tumor suppressors, the depth and quantity of the publicly available genome-wide data are much smaller than those in genomic alterations and expression profiling studies. Therefore, we utilized the data of genomic copy number alterations and mRNA expression levels of GBM, selected the candidate genes, and screened through *in vivo* RNAi screen. Tumor suppressors are pivotal modulators of cancer genetics, and it is likely that many of “tumor suppressor” genes may not have inactivating point mutations as a primary cause for functional inactivation. There is a significant lack of functional validation of these candidate genes. The present study represents a step towards this goal. Technical feasibility of *in vivo* RNAi screen in patient-derived primary GBM models shown here suggests that this approach can be also applicable to the search of potential oncogenic effectors in GBM.

NLK is a serine/threonine protein kinase that regulates diverse signaling processes via phosphorylation of several transcription factors [27, 28, 42–44]. We and others have previously shown that aberrant activation of WNT pathway promotes multiple aspects of GBM biology including tumor initiation, proliferation, inhibition of apoptosis, and stem-like features [32, 33, 45, 46]. Despite the prominent roles of WNT pathway in GBM, GBM rarely have mutations in the negative regulators such as APC and NLK. Notably, a subset of GBMs with the lowest NLK expression harbors very high levels of both WNT and mesenchymal activities. It is possible that NLK loss is a causative event in aberrant WNT activation at least in this subset of GBMs. Further studies to investigate GBM evolution process are warranted.

Our data indicate NLK loss in GBM is not completely restricted to mesenchymal subtype. Indeed, we found weaker correlations between NLK loss and the signaling pathways such as TGF-beta, NF-κB, and STAT3, compared to WNT pathway. Mesenchymal features can be driven by multiple factors including tumor genetics and microenvironment. The proneural and the mesenchymal subtypes have been defined as the most consistent subgroups in GBM by transcriptome analysis [6, 36, 37]. However, more recent studies have reported proneural to mesenchymal switching and co-existence of multiple subtypes within a GBM, suggesting a dynamic nature of GBM subtype [35, 36, 47, 48]. In this context, causative roles of NLK loss in mesenchymal properties of GBM can be more prominent in a subset of GBMs.

In conclusion, our studies demonstrate *in vivo* RNAi screening tool as a powerful tool to identify and validate a candidate tumor suppressor. Currently, most WNT pathway targeted drugs have been developed against the ligands, receptors, and an intermediate signaling molecule GSK3β. Given the profound effects of NLK overexpression in inhibition of WNT, mesenchymal, and stem cell associated proteins, it is conceivable to develop therapeutic approaches that mimic the function of NLK. Furthermore, NLK level can be a biomarker to predict WNT and/or mesenchymal activities in GBM for the selection of patient groups that are most likely to be benefit from WNT pathway targeting therapy.

## MATERIALS AND METHODS

### RNAi screen

For shRNA screen procedure, different sets of cells were infected with a pool of approximately 200 lentiviral shRNAs targeting 24 human genes at a representation of ~ 500 cells per shRNAs at Multiplicity of Infection at 1. On day 2 of the post infection, puromycin (Sigma) (1ug/ml) was added to remove any non-infected cells and the selection procedure proceeded for the next 3 days. Afterwards, 100,000 transduced cells were injected into its recipient mice and the Control populations were harvested. For each corresponding samples, shRNA barcodes were PCR-recovered from genomic samples, and analyzed through deep sequencing technology (Illumina High-Seq 2000). Each shRNA read was normalized to its whole population and changes in the relative abundance of each shRNA in the library were measured.

### Patient-derived GBM specimens and primary cell culture

Following informed consent in accordance with the appropriate Institutional Review Boards, glioblastoma specimens were obtained from patients undergoing surgery. Patient-derived GBM cells were cultured in the “NBE” neurosphere culture condition [21]

### Orthotopic GBM xenograft models

Six-week-old female BALB/c nude mice (Orient Bio, Seoul, South Korea) were used for intracranial injection. Patient-derived GBM cells were injected into the brains of mice by stereotactic intracranial injection (coordinates: 2 mm anterior, 2 mm lateral, 2.5 mm depth from the dura). Mice were killed either when 25% body weight loss or neurological symptoms (lethargy, ataxia, and seizures) were observed. All mice experiments were performed according to the Association for Assessment and Accreditation of Laboratory Animal Care-accredited guidelines of our institute's Animal Use and Care Committee.

### TMA and tumor samples

For analysis of NLK levels by immunohistochemistry, TMA containing glioblastoma and normal brain tissues were used. The TMA slides contain 88 cases of glioblastoma and 31 of their own accordant normal brain tissue parts. Brain tissue samples were fixed by formalin and embedded in paraffin; then sections of paraffin-embedded glioma specimens were stained with an antibody against human NLK (Sigma-Aldrich), CD44 (Sigma-Aldrich), and YKL40 (Life-Science).

### Lentivirus production and transduction

To generate recombinant lentivirus, a cloning package including entry (pCR8/GW/TOPO) and lentiviral destination (pLenti6/V5-Dest) vectors were used (Invitrogen). 293FT cells were transfected using lipofectamine 2000 (Invitrogen) for the production of lentiviruses. After the initial transfection, supernatant was collected after 24, 48, 72 hours respectively and were concentrated. For the transduction of GBM cells, lentiviruses were added into the culture medium for 2 days and then blasticidin (3~4 ug/ml) selection was performed to eliminate any non-infected cells.

### Quantitative real-time RT-PCR

RNAs were extracted (Qiagen) and their complementary DNAs were synthesized (Invitrogen) per manufacturers'’ instructions. Real-time RT-PCR was performed using primers listed in Supplementary Table 2 (7500 Fast Real-Time PCR System). Duplicate reactions were performed for each set of primers and the relative amounts of target transcripts were normalized to the number of human beta-actin transcripts. The relative quantification of target gene expression was performed with the comparative cycle threshold (CT) method.

### Cell proliferation assay and neurosphere forminglimiting dilution assay

Cell proliferation was measured using EZ-cytox cell viability kit (DAEIL Lab, Seoul, South Korea) according to the manufacturer's protocol. A total of 1 × 104 cells were plated per well in a 96-well plate, and each sample were plated in quintuplicate. After 7 days, Ez-cytox was added into each well accordant to its appropriate volume and incubated for three hours. At the end of the incubation, cell viability was evaluated by measuring the optical density at 450nm. For neurosphere forming limiting diluation assay, cells were seeded at a range of 1–200 cells per well. After 1~2 weeks, the number of wells without spheres were counted. LDA clonogenic index was calculated as the inverse of the x-intercept of the regression between the number of wells without spheres and the number of cells seeded.

### Immunofluorescence analysis

Cells were fixed in 4% paraformaldehyde in PBS for 20 minutes and blocked with 5% donkey serum. Afterwards, they were incubated with primary antibodies over night at 4°C and then with anti-mouse Alexa 594 (Invitrogen) and anti-rabbit Alexa 647 (Invitrogen) secondary antibodies for 2 hrs with washing in between. Microscopy was done with a confocal microscope.

### Western Blot assay

Cells were lysed in RIPA buffer supplemented with proteinase inhibitor and phosphatase inhibitor. Total proteins (15~30 μg/lane) were separated by SDS-PAGE and transferred to PVDF membranes (Millipore). The blots were blocked for 1 hr in 5% Bovine Serum Albumin in TBS-T and were incubated with mouse anti-V5 NLK (Invitrogen), NLK (Sigma-Aldrich) Sox2 (R&D Systems), Nanog (Cell Signaling), LEF1 (Cell Signaling), C-myc(Cell Signaling), p-LEF1 (Millipore), anti-FLAG (Cell Signaling), and Cyclin D1 (Cell Signaling) overnight at 4°C. After washing with TBS-T, the blots were incubated with HRP-conjugated secondary antibody for 1 hr at room temperature. Detection was performed using the chemiluminescence method (ECL, GE Healthcare).

### Luciferase assay

GBM cells from a reporter tumor were transduced with appropriate lentivirus. After 2 days, blasticidin was added to eliminate any uninfected cells. Post 3 days of blasticidin (Invitrogen) selection, total cell extracts were prepared using GloLysis Buffer, and the luciferase activities were analyzed using the Steady-Glo Luciferase Assay System accordance with the manufacturer's protocol (Promega, USA). Each value was normalized to its total protein amounts and TCF/LEF luciferase value was normalized to mCMV backbone luciferase value.

### Flow cytometry

Cells were fixed and permeabilized using solutions from BD Biosciences. They were stained with different primary antibodies with their accordance purposes; CD44 (Sigma-Aldrich), and Annexin V (eBioscience). Afterwards, they were stained with Alexa 647 (Invitrogen) secondary antibodies. Flow cytometry was performed in FACS Calibur (BD Biosciences).

### Chromatin immunoprecipitation (ChIP) assay

Chromatin immunoprecipitation (ChIP) was carried out using EZ ChIP kit (Millipore) following the manufacturer's protocol. Briefly, the cells were formaldehyde crosslinked and the lysates were shared by ultrasonication and cleared by centrifugation and diluted in ChIP dilution buffer. IP complexes were immunoprecipitated with LEF1 antibody (Santa Cruz), normal Goat IgG (negative control) and RNA pol II (positive control). Crosslinks were reserved by incubating chromatin at 65°C overnight and enriched DNA was amplified by PCR and primers indicated below.

CD44 promoter forward, 5′ – CTGCGTTTGATTTCCAAACA – 3′ and

CD44 promoter reverse, 5′ – CCTACCCAGCAGATCTTAAAGAGAGG – 3′,

YKL40 promoter forward, 5′ – CTGTTCACCCCTCCCCTAACACT – 3′

YKL40 promoter reverse, 5′ – GGCTGAAAATCTGTCTATTCTTCTG – 3′.

### Statistical analysis

All statistical analysis was conducted using Student's *t* test to determine the significance of results. Overall survival curves were plotted according to the Kaplan-Meier method. All differences were considered to be statistically significant at the level of *P* < 0.05, *P < 0.01, **P < 0.001; ***

## SUPPLEMENTARY FIGURES AND TABLES


