# Supplementary material for: In vivo RNAi screen identifies NLK as a negative regulator of mesenchymal activity in glioblastoma
Source: Oncotarget. 2015 May 19;6(24):20145–59. doi: 10.18632/oncotarget.3980 (PMC4652994; doi:10.18632/oncotarget.3980)
Supplement: Supplementary file 1 [file oncotarget-06-20145-s001.pdf]

SUPPLEMENTARY FIGURES AND TABLES

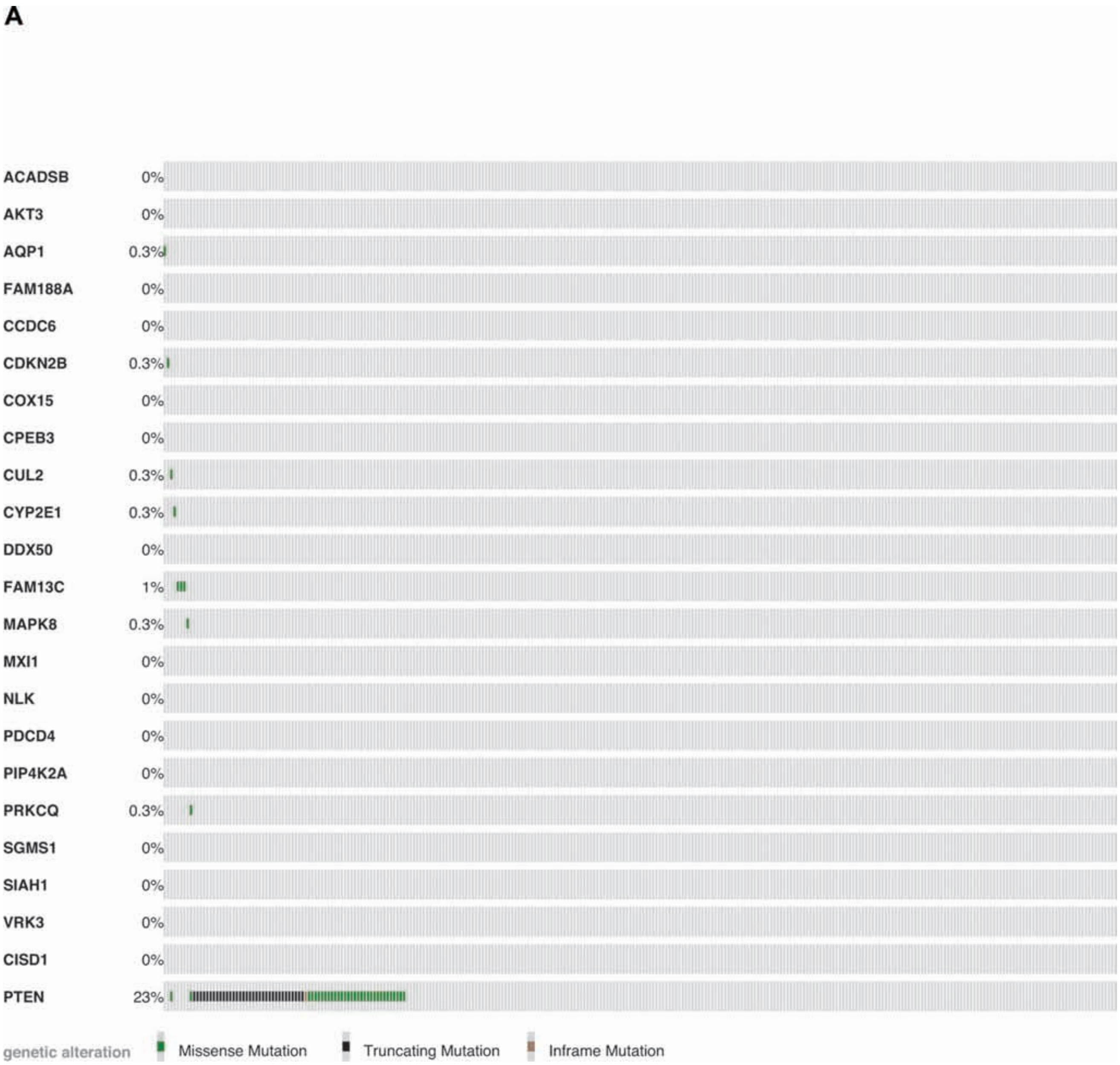

Supplementary Figure S1: A list of reported frequency of somatic mutations in GBM. A. Reported frequency of somatic mutations of our candidate tumor suppressor genes that are found in TCGA GBM from cBioportal public database.

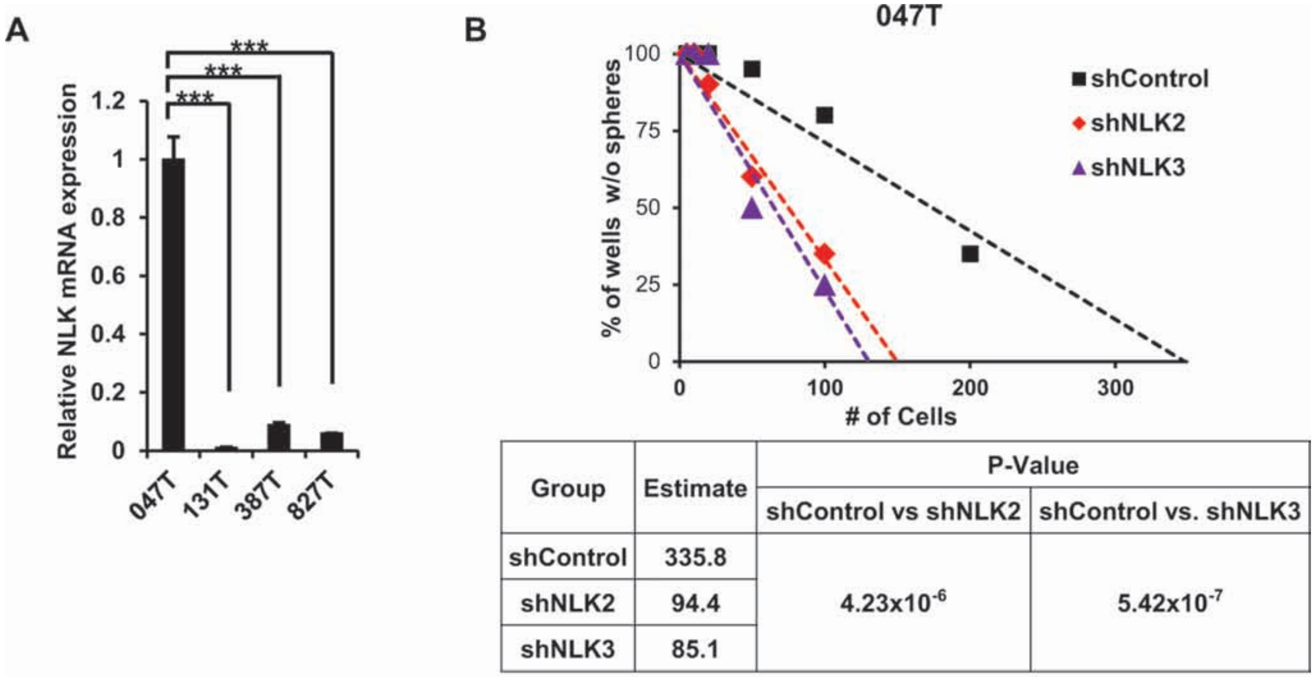

Group	Estimate	P-Value	
		shControl vs shNLK2	shControl vs. shNLK3
shControl	335.8	4.23×10^{-6}	5.42×10^{-7}
shNLK2	94.4		
shNLK3	85.1		

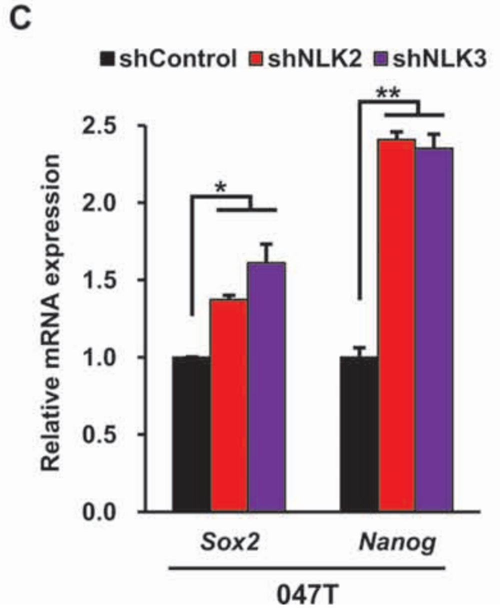

Supplementary Figure S2: Proliferation and clonogenic growth of GBM cells in the presence of shRNA-mediated NLK knockdown. A. Real-time RT-PCR analysis of NLK mRNA expression in different GBM cells. **B.** Limiting dilution assays (LDA) for *in vitro* tumor sphere formation. Primary GBM tumor cells were transduced with shControl, shNLK2, or shNLK3 vectors and plated into 96-well plates with various seeding densities (1–50 cells per well) for sphere formation. LDA clonogenic significance is measured by the linear regression analysis. **C.** Real-time PCR analysis of stem cell associated transcription factors (Sox2 and Nanog).

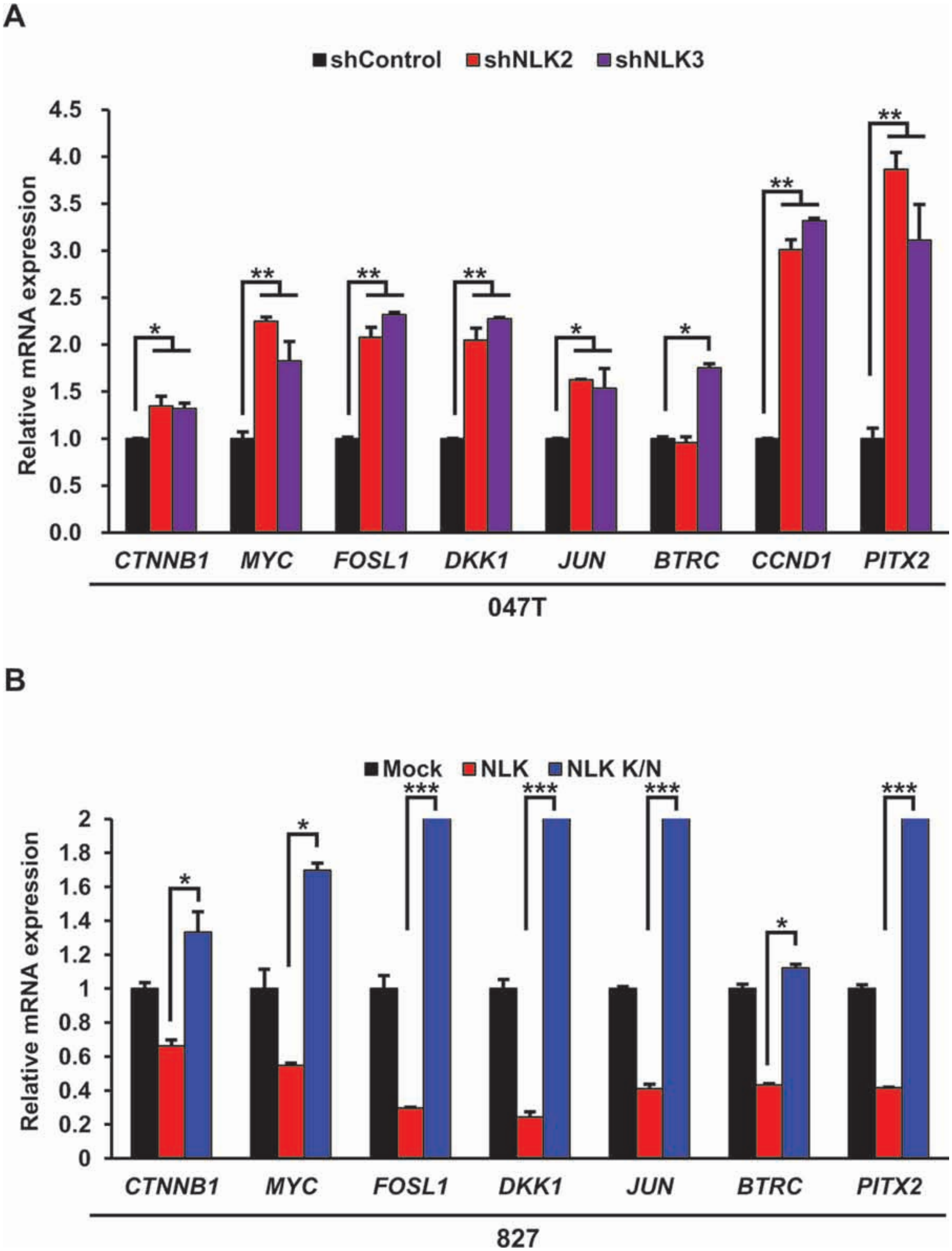

Supplementary Figure S3: Analysis of Wnt target genes. A. Real-time PCR analysis to determine the effects of shNLK on mRNA expression levels of beta-catenin and its downstream target genes (*MYC*, *FOSl1*, *DKK1*, *JUN*, *BTRC*, and *PITX2*), (+ SD, *n* = 2). **B.** Real-time PCR analysis to determine the effects of NLK K/N on mRNA expression levels of beta-catenin and its downstream target genes (*MYC*, *FOSl1*, *DKK1*, *JUN*, *BTRC*, and *PITX2*), (+ SD, *n* = 2).

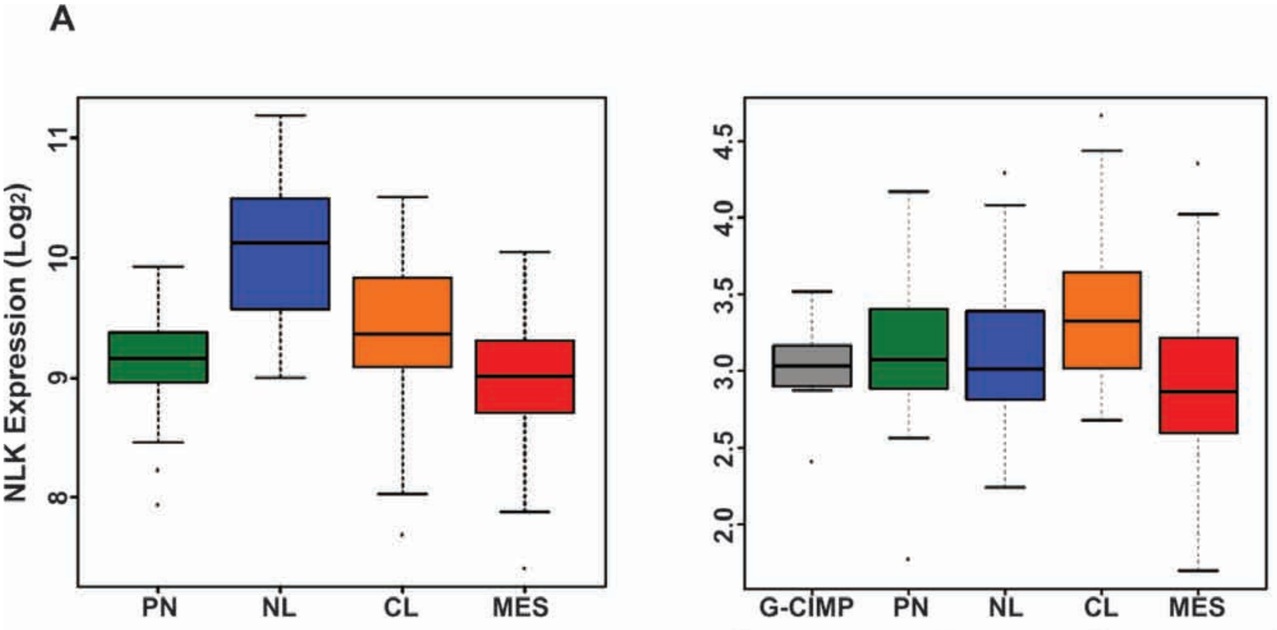

Subtype	N	P.Value
Proneural	49	1.34E-01
Neural	48	7.84E-18
Classical	52	2.13E-04
Mesenchymal	63	1

Subtype	N	P.Value
G-CIMP	38	2.93E-01
Proneural	99	5.31E-04
Neural	62	2.45E-02
Classical	128	1.32E-09
Mesenchymal	162	1

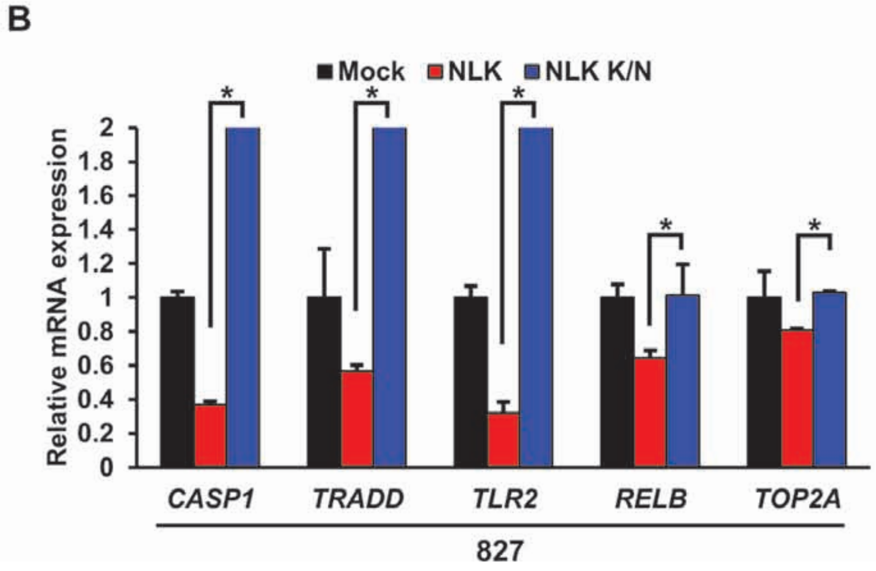

Supplementary Figure S4: GBM subtype classification according to NLK mRNA expression. A. Rembrandt microarray data analysis of NLK expression according to four different GBM subtype classification. (PN: Proneural, NL: Neural, CL: Classical, MES: Mesenchymal) and TCGA RNA-seq data analysis of NLK expression according to different GBM subtype classification and G-CIMP group. **B.** Real-time PCR analysis to determine the effects of NLK K/N on mRNA expression levels of mesenchymal associated genes (CASP1, TRADD, TLR2, RELB, TOP2A), (+ SD, *n* = 2).

Supplementary Table S1. A list of the shRNA pool

A. List of shRNA target genes that were used in our *in vivo* RNAi screen.

Gene Symbol	Location	Gene Full Name
ACADB	10q26.13	Acyl-CoA dehydrogenase, short/branched chain
AKT3	1q44	v-akt murine thymoma viral oncogene homolog 3 (protein kinase B, gamma)
AQP1	7p14	Aquaporin 1
FAM188A	10p13	Family with sequence similarity 188, member A
CCDC6	10q21	Coiled-coil domain containing 6
CDKN2B	9p21	Cyclin-dependent kinase inhibitor 2B (p15, inhibits CDK4)
COX15	10q24	COX15 homolog, cytochrome c oxidase assembly protein
CPEB3	10q23.32	Cytoplasmic polyadenylation element binding protein 3
CUL2	10q11.21	Cullin 2
CYP2E1	10q23.32	Cytochrome P450, family 2, subfamily E, polypeptide
DDX50	10q22.1	DEAD (Asp-Glu-Ala-Asp) box polypeptide
FAM13C	10q21.1	Family with sequence similarity 13, member C
MAPK8	10q11.22	Mitogen-activated protein kinase 8
MXI1	10q24-q25	MAX interactor 1
NLK	17q11.2	Nemo-like kinase
PDCD4	10q24	Programmed cell death 4 (neoplastic transformation inhibitor)
PIP4K2A	10p12.2	Phosphatidylinositol-5-phosphate 4-kinase, type II, alpha
PRKCQ	10p15	Protein kinase C, theta
PTEN	10q23.3	Phosphatase and tensin homolog 1
SGMS1	10q11.2	Sphingomyelin synthase 1
SIAH1	16q12	Seven in absentia homolog 1
VRK3	19q13	Vaccinia related kinase 3
CISD1	10q21.1	CDGSH iron sulfur domain 1

Supplementary Table S2. A list of Primer Sequences

A. List of all the primer sequences that were used for real-time PCR.

Real-Time PCR Primers	Sequence
Sox2 Forward	5′ – TGT AGA TTG CAG GCA GAC AGA - 3′
Sox2 Reverse	5′ – TGG GAA GAA GAT CAC GAA G – 3′
Nanog Forward	5′ – CTA AGA GGT GGC AGA AAA ACA - 3′
Nanog Reverse	5′ – CTG GTG GTA GGA AGA GTA AAG G - 3′
CTNNB1 Forward	5′ – CAT CTA CAC AGT TTG ATG CTG CT - 3′
CTNNB1 Reverse	5′ – GCA GTT TTG TCA GTT CAG GGA - 3′
MYC Forward	5′ – GGC TCC TGG CAA AAG GTC A - 3′
MYC Reverse	5′ – CTG CGT AGT TGT GCT GAT GT - 3′
FOSL1 Forward	5′ – CAG GCG GAG ACT GAC AAA CTG – 3′
FOSL1 Reverse	5′ – TCC TTC CGG GAT TTT GCA GAT – 3′
DKK1 Forward	5′ – CCT TGA ACT CGG TTC TCA ATT CC – 3′
DKK1 Reverse	5′ – CAA TGG TCT GGT ACT TAT TCC CG – 3′
JUN Forward	5′ –TCC AAG TGC CGA AAA AGG AAG – 3′
JUN Reverse	5′ – CGA GTT CTG AGC TTT CAA GGT – 3′
BTRC Forward	5′ – CCA GAC TCT GCT TAA ACC AAG AA – 3′
BTRC Reverse	5′ – GGG CAC AAT CAT ACT GGA AGT G - 3′
PITX2 Forward	5′ – CGG CAG CGG ACT CAC TTT A – 3′
PITX2 Reverse	5′ – GTT GGT CCA CAC AGC GAT TT – 3′
CASP1 Forward	5′ – TTT CCG CAA GGT TCG ATT TTC A – 3′
CASP1 Reverse	5′ – GGC ATC TGC GCT CTA CCA TC – 3′
TRADD Forward	5′ – GCT GTT TGA GTT GCA TCC TAG C – 3′
TRADD Reverse	5′ – CCG CAC TTC AGA TTT CGC A – 3′
TLR2 Forward	5′ – ATC CTC CAA TCA GGC TTC TCT – 3′
TLR2 Reverse	5′ – GGA CAG GTC AAG GCT TTT TAC A – 3′
RELB Forward	5′ – CCA TTG AGC GGA AGA TTC AAC T – 3′
RELB Reverse	5′ – CTG CTG GTC CCG ATA TGA GG – 3’A
TOP2A Forward	5′ – ACC ATT GCA GCC TGT AAA TGA – 3′
TOP2A Reverse	5′ – GGG CGG AGC AAA ATA TGT TCC – 3′
